# Leukemoid reaction in cervical cancer: a case report and review of the literature

**DOI:** 10.1186/1471-2407-14-670

**Published:** 2014-09-15

**Authors:** Li Qing, Tao Xiang, Zhang Guofu, Feng Weiwei

**Affiliations:** Department of Gynecology, Obstetrics and Gynecology Hospital, Fudan University, Shen Yang Road 128, Shanghai, China; Department of Pathology, Obstetrics and Gynecology Hospital, Fudan University, Shanghai, China; Department of Radiology, Obstetrics and Gynecology Hospital, Fudan University, Shanghai, China; Shanghai Key Laboratory of Female Reproductive Endocrine-Related Disease, Fudan University, Shanghai, China

**Keywords:** Leukemoid reaction, Cervical cancer

## Abstract

**Background:**

The presentation of a leukemoid reaction in cervical cancers is rare. A leukemoid reaction is defined as leukocytosis associated with a cause outside the bone marrow, with the white blood cell count (WBC) exceeding 50*10^9^/L. Two cervical cancers presenting with leukemoid reactions were previously reported. However, the cancers in these cases were mainly in the advanced stages and had a poor outcome.

**Case presentation:**

Here we report a 40-year old patient with clinical stage (FIGO IIA1) cervical squamous carcinoma suffering from vaginal cuff recurrence with a leukemoid reaction two months after laparoscopic radical hysterectomy. The patient suffered from persistent fever and leukocytosis after one month of antibiotic treatment accompanied by rapid growth of the vaginal mass indicated that the leukocytosis could not be caused only by infection. After paclitaxel injection, the WBC count increased to 70.37*10^9^/L. Bone marrow aspirates and biopsy showed left-shift neutrophilia, which confirmed leukemoid reaction. After two courses of paclitaxel and cisplatin treatment, the white blood cell counts decreased to normal, the fever disappeared, and the vaginal mass was reduced dramatically. She achieved completed remission after subsequent chemo-radiation and two additional courses of chemotherapy.

**Conclusion:**

In our case, leukemoid reaction was related to recurrent cervical carcinoma and sensitive to chemotherapy. To our knowledge, this is the third case to be reported in the literature. Furthermore, this is the only case described that shows an unequivocal correlation between tumor response and leukemoid reaction.

## Background

Cervical cancer is the third most common cancer and the fourth leading cause of cancer death in women worldwide, and more than 85% of cases occur in developing countries [1]. Clinically, the primary treatment for early stage cervical cancer is surgery, radiation therapy, or concurrent chemoradiation. The 5-year survival rate for early stage cervical cancer is 70% [2].

A white blood cell count (WBC) exceeding 50*10^9^/L associated with a cause outside the bone marrow is termed a leukemoid reaction (LR). LR is generally benign, although it could resemble more serious conditions that are medical dilemmas. Its frequency in patients with nonhematologic cancers remains unclear, although it has been reported to range from 1% to 4% in small case series. LR has been reported in nearly all solid tumor types as a paraneoplastic syndrome and is historically associated with poor outcome. Cervical cancers presenting with LR are rarely reported. Only two authors, Nimieri [3] and Kyo [4], have reported LR in cervical cancer patients. However, the cancers in these cases were mainly in advanced stage. Here, we report a patient with relatively early stage (International Federation of Gynecology and Obstetrics, FIGO IIA1) cervical squamous carcinoma suffering from recurrence with LR two months after laparoscopic radical hysterectomy. The clinical and histological features are described. The literature describing these rare cases is also reviewed.

## Case presentation

### Case report

**Figure 1:**
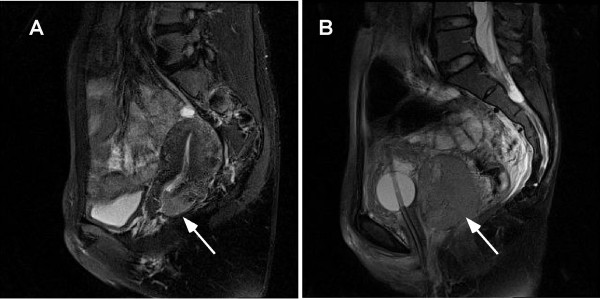
**Magnetic resonance imaging of primary and recurrent cervical mass. A.** The arrow indicated a 2.7*2.2*3.4 cm lesion in the posterior cervix, accompanied by disappearance of the posterior fornix. **B.** The arrow indicated a 68*63*58 mm mass in the vaginal cuff.

**Figure 2:**
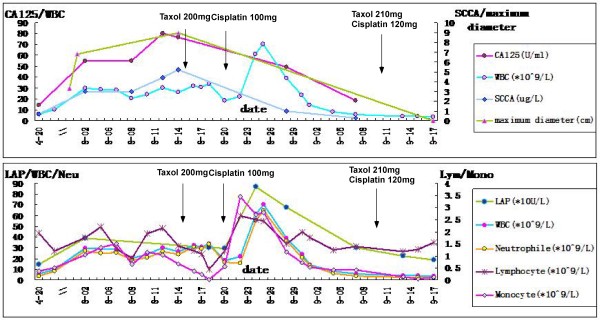
**Dynamic changes in tumor volume (the maximum diameter of tumor), tumor markers (SCCA and CA125), white blood cell (WBC) counts, and LAP response to chemotherapy. Upper panel:** The levels of CA125 and SCCA were correlated with the tumor volume. The WBC counts remained at a high level since recurrence and reached its highest value after the first round of chemotherapy. **Bottom panel:** The levels of LAP, neutrophils, lymphocytes, and monocytes were in accordance with the WBC counts.

**Figure 3:**
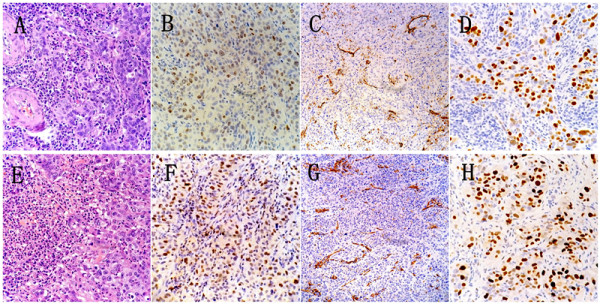
**HE and immunohistochemical (Ki-67, CD31, P53) staining of primary (A, B, C, D) and relapsed (E, F, G, H) cancer tissues.** H&E staining **(A)** showed irregular nests of squamous cell carcinoma dispersed in the stroma, with various quantities of inflammatory cells infiltrating the stromal tissues as well as cancerous nests. In the relapsed cancer tissue **(E)**, neutrophils were more predominant, whereas in primary cancer tissues, a mixed population of lymphocytes and neutrophils was observed. The immunohistochemical staining patterns of Ki-67 **(B, F)**, CD31 **(C, G)**, and p53 **(D, H)** were similar in the primary and relapsed tissue. Ki-67 positively expressed in >90% of tumor cells. P53 was strongly expressed in tumor cells.

A 40-year-old woman who was experiencing increased discharge and postcoital bleeding for 2 months was admitted to the Obstetrics and Gynecology Hospital of Fudan University on April 18, 2012. A 3.5 cm cervical stump was found via bimanual examination, and tissue biopsy results verified that it was infiltrative cervical squamous carcinoma. Magnetic resonance imaging (MRI) examination indicated a 2.7*2.2*3.4 cm lesion in the posterior cervix, accompanied by disappearance of the posterior fornix. (Figure 1A) Preoperative examinations, including a routine blood test (WBC: 5.93*10^9^/L, neutrophils: 58%, lymphocytes: 33%, monocytes: 6%, eosinophils: 2%), analysis of tumor markers (squamous cell cancer antigen, SCCA: 0.7 ng/mL; CA125: 14.32 U/L), and determination of the leukocyte alkaline phosphate score (LAP: 145 U/L), produced normal results (Figure 2). Because the patient was diagnosed with Federation of Gynaecology and Obstetrics (FIGO) IIA1 cervical cancer, she underwent surgery (laparoscopic radical hysterectomy, pelvic lymphadenectomy, vaginoplasty, and ovarian transposition) on April 24, 2012. A pathological examination revealed a 3 cm non-keratinized squamous carcinoma located in 3°-9° of the cervix, with distances of 1.5 cm up to internal orifice and 0.5 cm down to the fornix. The tumor was confined to the inner half of the cervical stroma; lymphovascular invasion and pelvic lymph node metastasis were not detected. Immunohistochemical staining showed positivity for p16 (+++), p53 (+), and Ki-67 (90%+), and CD31 and D240 staining confirmed that the vessels were intact (Figure 3). Because the patient had no risk factors for adjuvant therapy, she was dismissed from the hospital 7 days after the operation.

**Figure 4:**
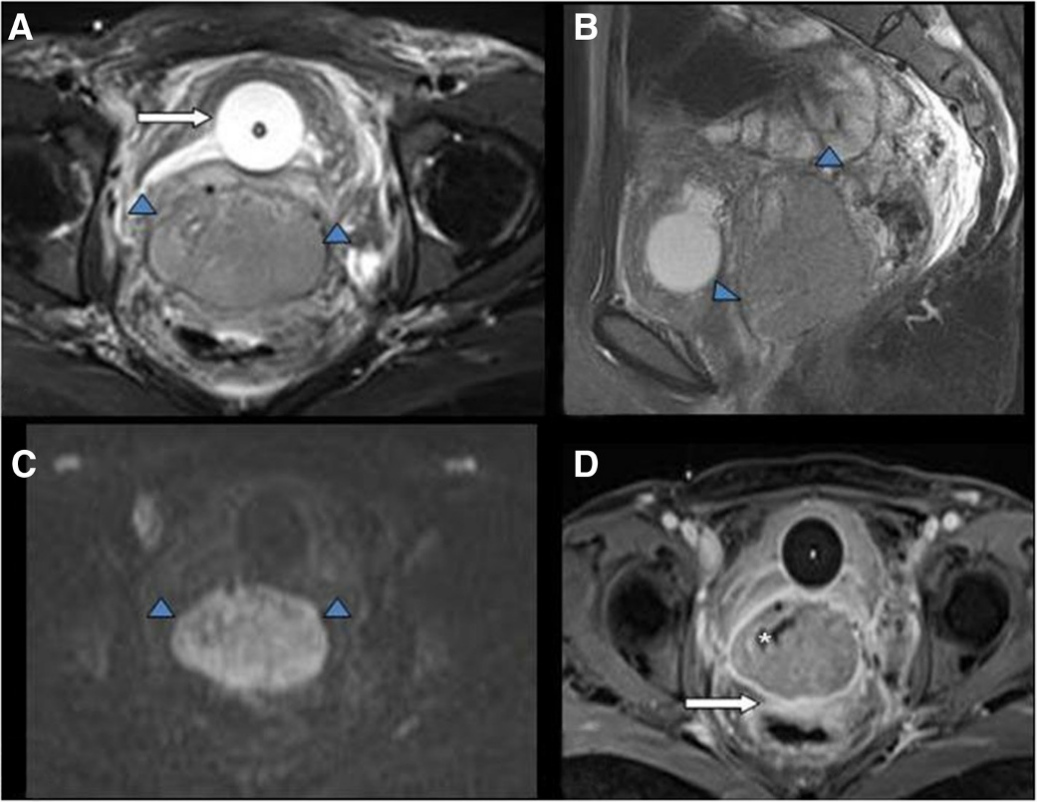
**MRI scans for the recurrent tumor. (A)** Axial fat-suppressed T2WI; **(B)** Sagittal fat-suppressed T2WI; **(C)** Diffusion weighted imaging (DWI); **(D)** Contrast-enhanced fat-suppressed T1WI. In this case, the giant oval mass (arrowhead) with homogeneous isointensity signal was well demonstrated on T2WI **(A, B)**. Note, the balloon catheter (arrow) was placed into the bladder. On DWI **(C)**, the tumor (arrowhead) appears hyper-intensity signal compared with hypointensity signal of background. On contrast-enhanced MRI **(D)**, the tumor mildly enhanced and the necrosis component (*) did not enhanced. Note, the mass-rectum margin was not clear (arrow), indicating the tumor invaded the anterior wall of rectum.

On July 2, 2012 (68 days after the operation), she presented to the outpatient clinic with increasing discharge. A 2*3 cm swollen cystic area was found in the posterior vagina. As purulent cells were found in her vaginal discharge, she was diagnosed with vaginitis and prescribed ornidazole to control the infection. On July 16, 2012, due to a high fever of 39.2°C and urine retention, she went to the urological outpatient clinic to receive catheterization. With a white blood cell count of 23*10^9^/L, she was diagnosed with a urinary tract infection and received intravenous cefotiam and levofloxacin. However, her fever was not well controlled. On July 30, 2012, a 60 mm diameter solid mass on the posterior vagina with an ulcerous surface was found by gynecological examination. MRI scan confirmed a homogeneous solid 68*63*58 mm mass located in the space between the bladder and the rectum (Figures 1B and 4). MRI well displayed the tumor itself and its margin. After injection of contrast materials, the relationship between the tumor and surrounding tissues also was well appreciated on serial MRI scans. Hydronephrosis of both kidneys and distension of upper region of the left ureter were confirmed by ultrasonic inspection. Biopsy was performed on the mass, which demonstrated degenerated squamous cells within necrotic tissue.

She was admitted to the inpatient ward again on August 2. 2012. Because of an uncontrollable fever (range: 39–40.5°C) and leukocytosis (WBC: 29.66*10^9^/L), samples of her blood, urine, and vaginal discharge were taken for bacterial culture to identify the source of infection. The cultures were all negative on August 2, 2012; however, Escherichia Colli, ESBLs (extended-spectrum β-Lactamase) sensitive to tienam and amikacin were found in a vaginal bacterial culture on August 5, 2012. Repeated discharge culture on August 12, 2012 was negative and became ESBLs positive again on August 17, 2012, then turned to negative on August 29, 2012. Since admission, empiric therapy with ceftriaxone sodium and metronidazole had been used; however, this was subsequently changed to a combination of tienam and vancomycin according to a drug sensitivity test on Aug 5, 2012. From August 12, 2012 to August 24, 2012, the patient was treated with norvancomycin and cefoperazone-sulbactam and subsequently with piperacillin-tazobactam plus amikacin for an additional 7 days.

On the other hand, although a biopsy on July 30, 2012 showed no malignancy (the possible negative result might be attributed to superficial biopsy sample), the recurrence of cervical carcinoma was highly suspected because the vaginal mass increased from 22*33*22 mm on July 2, 2012 to 63*65*58 mm on July 30, 2012 and the characteristics showed by MRI highly suggested a recurrent tumor. The tumor mass increased to 80 mm in diameter by palpation on August 6, 2012. A second bulky biopsy was performed on August 7, 2012. In order to get enough tissue, we cut the surface open, and excised a 60*40*20 mm mass. The whole mass was solid without abscess. After the biopsy, the tumor volume shrank to 70*40*40 mm. Pathology examination confirmed recurrent squamous carcinoma. Immunochemistry staining for Ki-67 and P53 was also performed (Figure 3). Chemotherapy was considered but was postponed because the patient was recently given sensitive antibiotics (tienam and vancomycin) and effective control of fever and leukocytosis was expected. However, one week later, the high fever (up to 39.2°C) and leukocytosis (up to 33.4*10^9^/L) were persistent. In addition, the mass continued growing to 90*82*74 mm on August 14, 2012 and tumor makers became abnormal (CA125: 76.66 U/L, SCCA: 5.3 ng/ml). Considering the severity of the situation, we decided to give the patient a new regimen of antibiotics and chemotherapy simultaneously.

Regarding chemotherapy, paclitaxel (200 mg) was administered intravenously on August 15, 2012. Unexpectedly, on the following day, her fever increased (40.3°C), and the planned cisplatin treatment was cancelled. On August 21, 2012, cisplatin (100 mg) was injected because the patient had become relatively stable (fever, 39°C; WBC, 18.95*10^9^/L). On August 25, 2012, her WBC count reached 70.37*10^9^/L, and a leukemoid reaction (LR) was suspected. Bone marrow aspirates and biopsy showed an increased myeloid/erythroid ratio, active myeloid proliferation, and normal megakaryocytopoiesis. Meanwhile, her LAP level was extremely high (869 U/L). Considering the lab tests, the diagnosis of LR was established. On August 28, 2012 (12 days after paclitaxel and 1 week after cisplatin injection), her WBC count was reduced to 39.23*10^9^/L, the mass was reduced to 65*46*40 mm, and SCCA and CA125 had decreased to 1.0 ng/ml and 49.46 U/L, respectively. On September 4, 2012 (three weeks after paclitaxel treatment), her fever and WBC count decreased to normal levels (36.8°C and 8.1*10^9^/L). Subsequently, the levels of tumor markers returned to normal. The second course of paclitaxel (210 mg) and cisplatin (120 mg) was administered on September 10, 2012 and the vaginal mass was successfully eliminated on September 17, 2012. Additionally, after chemotherapy was performed, ultrasound results indicated that the kidney and IVP were normal. The dynamic changes in WBC, LAP, tumor makers, and tumor volume along with the chemotherapy are illustrated in Figure 2.

From October 10, 2012 to November 16, 2012, the patient received external irradiation to a dose of 45 Gy in 25 fractions with concurrent cisplatin chemotherapy. No abnormality was observed on MRI on November 19, 2012. After radiotherapy, she was treated with chemotherapy (100 mg docetaxel together with 300 mg carboplatin) on December 17, 2012 and January 14, 2013. The follow-up was satisfying: her WBC, CA125, and SCCA levels were normal. The latest follow-up on May 23, 2014 showed no evidence of recurrence.

## Discussion

### Data analysis

As depicted in Figure 2, the basal WBC level increased from 5.93*10^9^/L preoperatively to 29.66*10^9^/L at 99 days after the operation. A high fever with a sharp increase in WBC indicated infection. However, the infection was not severe as it was superficial. Persistent fever and leukocytosis after one month of antibiotic treatment accompanied by rapid growth of the vaginal mass indicated that the leukocytosis could not be caused only by infection. In addition, the characteristics on MRI findings such as homogeneous isointensity signal on T2WI and hyper-intensity signal compared with hypointensity signal of background on DWI highly suggested recurrent tumor (Figure 4). Furthermore, during the second biopsy, the mass was solid but containing no abscess at all. Last, after paclitaxel injection, the WBC count increased to 70.37*10^9^/L. Bone marrow aspirates and biopsy showed left-shift neutrophilia, which confirmed LR and excluded chronic myelogenous leukemia (CML). LAP detection was consistent with the trend in WBC counts. Three weeks after the first course of paclitaxel and cisplatin treatment, the levels of CA125, SCCA, and WBC decreased to normal, the fever disappeared, and the vaginal mass was reduced dramatically. SCCA, defined as squamous cell carcinoma antigen, one of the most popular diagnostic tumor markers of cervical cancer, has a sensitivity of 67-100% and specificity of 90-96% in recurrent cervical squamous cervical cancer. The elevation of SCCA could also be occurred in psoriasis, eczema, and severe kidney disease. In our case, SCCA was elevated with the recurrence and subsequently decreased after chemotherapy, which was in accordance with tumor burden. Thus, in our case, LR was related to recurrent cervical carcinoma and sensitive to chemotherapy. The LR was distinguished from CML directly by bone marrow biopsy and by an inverse correlation between tumor response and leukocytosis as well as an increased LAP [5].

### The possible cause of LR in cancer patients

As in many other carcinomas, we considered LR as a paraneoplastic syndrome associated with cancer and attributed to increased cytokine production. Fever is an integral component of leukemoid reaction resulting from either release of endogenous pyrogens or due to necrotic-inflammatory phenomena of the tumor. Various studies have reported elevations in granulocyte colony-stimulating factor (G-CSF), granulocyte-macrophage colony-stimulating factor (GM-CSF), and interleukin-6 (IL-6) in tumors of the cervix, pancreatic cancer, hepatocellular carcinoma, and lung cancer [4, 6–8]. Animal experiments have demonstrated that interleukin-6 (IL-6) can sustain neutrophil/macrophage colonies *in vivo* and that, through its action on other immune cells, it can induce the production of several CSFs by the bone marrow [9]. Despite stimulating bone marrow to sustain leukocytosis, IL-3, GM-CSF, and G-CSF have been shown to stimulate the growth of clonogenic cells of some nonhematopoietic malignant cell lines *in vitro* [5]. Hurtado et al. found that when G-CSF was overproduced by tumor cells, it had a tendency to encourage the development of myeloid-derived suppressor cells (MDSC), which could travel to lymph nodes to prevent dendritic cell-primed T lymphocytes from eliminating tumor cells [10]. Therefore, consistent with the clinical presentation of our case, paraneoplastic granulocytosis is often associated with rapid tumor growth and poor clinical prognosis, although it resolves with the treatment of the underlying cancers. Especially when infection is not likely or not the only contributor, the possibility of tumor origin should be considered when patients present with an LR of unknown etiology. Nevertheless, drugs that induce LRs, such as methotrexate [11] and carbamazepine [12], should be avoided.

### The pathological characteristics of cancers with LR

Although we did not measure the G-CSF, GM-CSF, or IL-6 in the tumor cells, we compared the HE sections and several immunochemical markers in primary and recurrent tumors to investigate the possible factors related to the unusual presentation in our case. Based on Figure 2, there were more neutrophils in the tissue after the recurrence than before the recurrence, although the primary lesion had more neutrophils than usual. The infiltration of the tumor by neutrophil granulocytes and lymphocytes proved to be significant for prognosis and discrimination [13]. A large number of neutrophils and lymphocytes in a cancerous lesion may be a clue indicating the potential for rapid recurrence. Nevertheless, Carus et al. recently assessed tumor-associated CD66b(+) neutrophils and CD163(+) macrophages by immunohistochemistry in whole tissue sections of 101 FIGO IB and IIA cervical cancer patients and found tumor-associated neutrophil count was an independent prognostic factor for short recurrence free survival in localised cervical cancer [14]. The Ki-67 index, a marker of cell proliferation, is similarly associated with tumor recurrence and prognosis. A 90% positive Ki-67 score in a primary lesion might be an indicator of rapid recurrence. P53, which is often associated with poor prognosis, was also remarkably positively expressed in primary and recurrent tumors.

### Treatment strategies for patients with LR

Treatment strategies for cancer with LR are limited and are rarely reported in the literature. As Granger and Kontoyiannis reported in a retrospective, single-institution study, patients who survived longer than 1 year received effective antineoplastic therapy, chemotherapy, or surgery. However, these authors did not mention specific strategies [15]. The majority of malignant tumors with LR respond poorly to chemotherapy, and the patients die shortly after. For example, a lung cancer patient with LR treated with various drugs, including vinorelbine, cisplatin, docetaxel, pemetrexed, and gemcitabine died two weeks after starting third-line therapy [16]. In our case, we used paclitaxel combined with cisplatin to control the development of LR. Surprisingly, after two courses of chemotherapy, the very large recurrent tumor completely disappeared and the WBC count decreased to normal. Subsequently, the patient received concurrent chemo-radiation therapy and an additional two courses of docetaxel combined with carboplatin. The patient survived for longer than 20 months after recurrence.

### Prognosis of tumor patients with LR

Patients with LR always have a poor prognosis with a tumor burden and a poor condition. Granger and Kontoyiannis [15] analyzed the etiology and outcome of extreme leukocytosis in 758 nonhematologic cancer patients, including 4 gynecological cancers, 1 of which was cervical cancer. Patients diagnosed with an LR typically had neutrophil predominance (96%), radiographic evidence of metastatic disease (78%), and a poor prognosis. In total, 78% of the patients either died or were discharged to hospice within 12 weeks of their initial extreme leukocyte count. Ma drew a similar conclusion in malignant bone tumor patients [17]. In a comprehensive review of inflammatory malignant fibrous histiocytoma (IMFH) associated with LR or leukocytosis, the severity of LR was correlated with the time of death. Once the WBC reached 100*10^9^/L, the patients were not capable of tolerating any chemoradiation secondary to their unstable clinical condition [10]. Mabuchi also concluded that pretreatment leukocytosis was an independent prognostic factor in patients with cervical cancer [18]. In comparison with other two cases of cervical cancer with LR in the literature, despite the severe condition (high fever and rapid growth of the tumor), our patient tolerated and responded to chemotherapy, and eventually achieved complete remission (Table 1).

**Table 1:** **Pathoclinical features in three cases of cervical cancer with LR**

Case*	Author	Year	Age	Stage	Previous treatment	Time to recurrence	Recurrence site	Pathology	WBC (*10^9/L)	Treatment	Survival
1	Satoru [4]	2000	56	IB2	Radical hysterectomy, bilateral salpingo-oophorectomy, and pelvic lymphadenectomy	15 days	Right parametrial space	SCC^#^	58	Not mentioned	68 days after surgery, DOD^$^
2	Nimieri [3]	2003	81	IVB	Chemotherapy and local radiotherapy	N/A		SCC	93	Chemotherapy, local radiotherapy	6 weeks, DOD
3	Current report	2013	40	IIA1	laparoscopic radical hysterectomy, pelvic lymphadenectomy, vaginoplasty, and ovarian transposition	68 days	Vaginal	SCC	70	Chemotherapy + radiation	20 months, Alive

*: A patient of cervical cancer with LR was mentioned in Granger and Kontoyiannis’s review article [14]. However, no information in detail was provided. Thus, this case was not included in Table 1.

^#^: SCC: Squamous cell carcinoma.

^$^: DOD: Dead of disease.

## Conclusion

To our knowledge, this is the first case report of an early stage cervical cancer suffering rapid recurrence with leukemoid reaction achieving a good prognosis due to early diagnosis and timely chemotherapy. Unusual infiltration of the tumor by neutrophil granulocytes and high expression of Ki-67 and P53 may relate to rapid recurrence and leukemoid reaction.

### Consent

Written informed consent was obtained from the patient for publication of this Case report and any accompanying images. A copy of the written consent is available for review by the Editor of this journal.
